# To-Day and Yesterday

**Published:** 1920-08-28

**Authors:** 


### FADS AND FASHIONS IN THERAPY.

### To-Day and Yesterday.

The fad is a motive force as uncontrolled by any form of logic as are the superstitions; and like these kindred powers, it plays a larger part in the common practices of to-day than many of us would be willing to admit. So long as it remains the source merely of individual action, the idea of the faddist may be properly regarded with amusement or perhaps with mild levity. Such an attitude, however, is no longer appropriate when the fad has spread so widely amongst the profession and the people as to constitute a fashion. For when this stage has been reached the fad will have become incorporated into the undefinable spirit of the herd and henceforth will be entirely free from all restraint of reason. Under the influence and impulse of custom or of fashion this herd spirit may lead the flock into absurd and foolish situations.

### Phlebotomy in the Past.

We may smile, nowadays, with the indulgence that our superior wisdom allows, at the monstrous excesses solemnly and sincerely committed by our forefathers in the performance of phlebotomy. Nevertheless, the fashion had serious consequences to the people of that generation, and many an individual was bled to death upon the altar of that fetish. It behoves us, therefore, to look around lest we too may be lending encouragement to fashionable tendencies which are as unreasonable as were the deeds of the old phlebotomists. For it does not require any great profundity to observe that the mentality which not only permitted, but wholeheartedly accepted, the crinoline as a desirable adjunct to a lady's costume is in fact the mentality of ourselves to-day. We still are capable of encouraging absurdities without reflection, provided that they have the sanction of fashion or of custom. And it is reasonable to suppose that future generations will be just as amazed at some of our existing practices as we ourselves are at those of our grandparents.

### Idols and Decay.

But it is never easy in trying to make a critical circumspection of our own times to cast our eyes upon the right spot. Usually it has been by a slow process of decay that the idols of mankind have crumbled down. Yet instances are not wanting in which their fate has been more suddenly determined by the onslaught of some shrewd observer whose eyes have penetrated their weakness and falsity.

Such an occasion it was when to Ambroise Paré, nearly four hundred years ago, were revealed the malefic effects of treating recent gunshot wounds with boiling oil. So prevalent was the custom, and so confident was Paré himself in its efficacy, that his adoption of gentler methods was due only to the lucky chance that he had run short of oil on an occasion when there were many wounded to be treated. Poor Paré had a sleepless night, fearing that in the morning he should find his patients dead from the poison of their wounds. He rose early, and, to his surprise, found that the wounds which had not been cauterised were in a much better condition than those which had been so treated. "Then I resolved," he tells us, "never more to burn thus cruelly poor men with gun-shot wounds."

### A Lesson to Heart.

Well, we may take such a lesson to heart and question some of our own methods. Not so very long ago patients with glycosuria were put to much misery by the enforcement of a strict, expensive, unsatisfying and unpalatable diet; not, be it noted, to relieve them from irritation brought about by the excessive excretion of sugar, but because it was the fashion. To-day we see that unkind fashion in process of decay. Other evil practices doubtless are in full swing, their harmfulness concealed by the veil of common custom. Indeed it is far easier to perceive and to criticise a fad, which is the larval forerunner of a fashion, than it is to form a sound opinion regarding a usage which has become generally prevalent.

### Psycho-Analysis.

Psycho-analysis, used as a therapeutic aid, appears to us in certain hands to be such a vagary. We do not deny the possible utility of the method in carefully selected cases, and as carried out by experienced men; but we do believe that the claims made upon its behalf by some individuals are so arrogant as to constitute a fad. Moreover it is one in which the public, attracted perhaps by its novelty and romantic possibilities, are beginning to take so much interest that the treatment is perilously near becoming a fashion. Enthusiastic doctors talk of giving up general practice in order to specialise in psycho-analysis. The subject is discussed by ladies in the boudoir. "Have you tried psycho-analysis?" a smart young woman was overheard to say; "I am trying it on my maids and I have done them some good already." Such gossip is but a straw, but it shows the direction of the wind. And a little contemplation will reveal much that is undesirable in the practice of psycho-analysis. Before we prise open a man's soul, surely we ought to be fairly sure that by such means alone we can give him help. It may be that by teaching him how to bury his dead past and lending him a hand in the work, we shall confer a greater and more lasting benefit than can be obtained merely by making him turn about and see for himself that the terror lying behind him is not a supernatural and paralysing incubus but merely a harmless corpse

